# What Drives Trust and Satisfaction in Health Welfare Social Cooperatives?

**DOI:** 10.3390/healthcare13121383

**Published:** 2025-06-10

**Authors:** Hyeon Jo, Enoch Kang, Bum Suk Lee

**Affiliations:** 1 Headquarters, HJ Institute of Technology and Management, 31 Gangnam-daero 92-gil, Gangnam-gu, Seoul 06134, Republic of Korea; sineoriz@gmail.com; 2 Department of Integrative Medical Humanities, Graduate School of Kyung Hee University, Gyeonghee-daero 26, Dongdaemun-gu, Seoul 02447, Republic of Korea; david@khu.ac.kr; 3 Department of Coaching Science, Graduate School of Business Kyung Hee University, Gyeonghee-daero 26, Dongdaemun-gu, Seoul 02447, Republic of Korea

**Keywords:** administrative procedures, collaborative management, community health relations, courtesy, physical accessibility, trust, satisfaction

## Abstract

**Background/Objectives**: Patient trust and satisfaction are critical components of effective healthcare delivery, particularly within health welfare social cooperatives. This study investigates the impacts of administrative procedures, courtesy, physical accessibility, collaborative management, and community health relations on patient trust and satisfaction. **Methods**: Using Partial Least Squares Structural Equation Modeling (PLS-SEM), we analyzed survey data collected from 658 members of a health welfare social cooperative to assess the hypothesized relationships. **Results**: The findings reveal that administrative procedures significantly enhance trust but do not directly affect satisfaction. Courtesy and community health relations positively influence both trust and satisfaction. Physical accessibility, collaborative management, and trust also significantly enhance patient satisfaction. **Conclusions**: These results highlight the multifaceted nature of patient trust and satisfaction, emphasizing the importance of efficient administrative practices, respectful treatment, collaborative decision-making, and strong community engagement. For practitioners, the study underscores the need for a comprehensive approach to healthcare management that integrates operational efficiency with patient-centered care.

## 1. Introduction

In contemporary healthcare, patient trust and satisfaction are pivotal components that determine the success and efficacy of health service delivery [1,2,3,4]. In this study, patient trust refers specifically to patients’ confidence in the clinical competence, reliability, and integrity of individual healthcare providers (e.g., doctors and staff), as well as their belief in the credibility and service quality of the health welfare social cooperative as a whole. This construct encompasses both interpersonal trust in medical professionals and institutional trust in the cooperative system, reflecting the multidimensional nature of trust within cooperative healthcare settings. Health welfare social cooperatives, operating on principles of mutual aid and collective ownership, offer a unique healthcare model [5]. These cooperatives involve members who pay contributions and actively participate in the cooperative’s management, influencing service delivery and perception [6]. Features include democratic decision-making, transparency in operations, and a focus on community health needs [7]. Understanding the factors driving patient trust and satisfaction within these cooperatives is essential for improving healthcare outcomes and ensuring the sustainability of these cooperative models. Enhancing these aspects can lead to better patient experiences, increased member engagement, and more effective healthcare services tailored to the community’s needs.

This study focuses on five key constructs—administrative procedures [8,9,10], courtesy [11,12,13], physical accessibility [8,14], collaborative management [6], and community health relations [6]—based on their relevance to patient-centered care in health welfare social cooperatives. Administrative procedures reflect the efficiency and transparency of healthcare delivery [8], while courtesy captures the interpersonal behavior and empathy shown by healthcare staff [11]. Physical accessibility refers to patients’ ease of reaching and navigating healthcare facilities [8]. Collaborative management represents the degree of shared decision-making and cooperative involvement in care processes [6]. Lastly, community health relations encompass how healthcare providers engage with and respond to the health needs of the broader community [6]. These constructs were selected as they collectively represent operational, relational, and systemic dimensions of healthcare quality that are highly relevant in cooperative models of care.

The healthcare sector has increasingly recognized the importance of administrative procedures in shaping patient experiences. Effective administrative procedures can enhance transparency, efficiency, and predictability, which are essential elements for building patient trust [8,15]. While some studies suggest that clear administrative processes can lead to improved patient satisfaction by reducing anxiety and streamlining service delivery [1], others argue that interpersonal interactions and the quality of clinical care may play a more significant role [16]. Courtesy, defined as respectful and empathetic treatment by healthcare staff, is another critical factor influencing patient perceptions. Previous research has established a strong link between courteous interactions and patient trust [11,17]. Courtesy not only enhances the perceived quality of care but also makes patients feel valued and respected, thereby increasing their overall satisfaction [18,19]. Physical accessibility, referring to the ease with which patients can access healthcare facilities, significantly impacts patient satisfaction. Accessibility issues can create barriers to timely and effective care, leading to dissatisfaction and poorer health outcomes [20,21]. Ensuring that healthcare facilities are conveniently located and equipped with adequate parking and accessibility features can reduce patient stress and improve their overall experience [22].

Collaborative management in healthcare involves the integration of diverse professional expertise and active patient participation in decision-making processes. This approach can enhance the perceived quality and comprehensiveness of care, thereby increasing patient satisfaction [23]. However, the impact of collaborative management on trust is less clear, particularly within health welfare social cooperatives where patients are already engaged in management activities [24]. Understanding how these collaborative practices influence both trust and satisfaction is important for optimizing management strategies in these cooperative settings. Community health relations, which involve the interaction and collaboration between healthcare providers and community organizations, play a vital role in enhancing service delivery and addressing local health needs [6,25]. Effective community health relations can create a supportive environment where patients feel more valued and cared for, leading to higher satisfaction levels [26]. This study aims to explore the significance of community health relations in shaping patient satisfaction within health welfare social cooperatives.

Trust and satisfaction are foundational constructs in healthcare service evaluation and play a central role in determining patients’ engagement, loyalty, and adherence to medical recommendations [27,28,29,30,31]. Patient trust reflects the belief that healthcare providers will act in the patient’s best interest with competence, honesty, and confidentiality [32,33]. It is essential not only for facilitating open communication but also for reducing uncertainty and anxiety in the care process [34]. Patient satisfaction, on the other hand, encompasses an individual’s overall evaluation of their healthcare experience, influenced by expectations, service delivery, interpersonal interactions, and perceived outcomes [35,36]. A strong body of research has shown that higher trust is consistently associated with greater patient satisfaction, increased compliance, and improved health outcomes [37,38,39]. These constructs are particularly relevant in cooperative healthcare contexts where participatory governance and long-term relationships with providers may further shape patients’ perceptions and expectations

Despite existing research on patient trust and satisfaction, two specific gaps remain: the lack of studies within the context of health welfare social cooperatives and insufficient examination of combined factors, such as medical quality, service quality, and social contribution. This paper addresses these gaps by investigating patient satisfaction within health welfare social cooperatives, using data from members across 13 national cooperatives, collected both online and offline. In this study, a national medical cooperative refers to a nonprofit, member-owned health organization that operates under cooperative principles across multiple regions in South Korea. These cooperatives aim to provide equitable, community-centered healthcare services, with members contributing financially and participating in governance and decision-making. The objective is to explain satisfaction in these unique settings. The originality and academic value lie in integrating multiple quality dimensions and providing a comprehensive analysis of patient satisfaction, contributing valuable insights to the field.

This study contributes to the theoretical understanding of trust and satisfaction by integrating underexplored dimensions such as community health relations and collaborative management into a unified framework tailored to cooperative healthcare settings. It also provides empirical evidence using structural equation modeling based on real-world data from health welfare social cooperative members across Korea. Practically, the findings offer actionable insights for healthcare managers and policymakers seeking to enhance trust and satisfaction through both service design and community engagement, especially in cooperative and community-based care models.

## 2. Literature Review and Research Model

**Figure 1:**
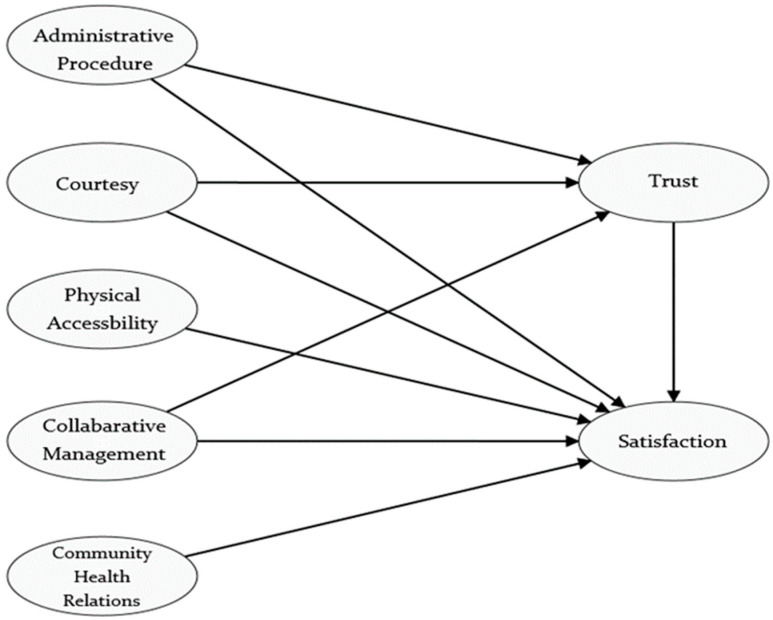
The Conceptual Framework.

The research model proposed in this study examines the effects of five key factors— administrative procedures [8,9,10], courtesy [11,12,13], physical accessibility [8,14], collaborative management [6], and community health relations [6]—on patient trust and satisfaction in the domain of healthcare services. Trust is modeled as both an outcome influenced by selected antecedents and a predictor of satisfaction, reflecting its central role in shaping patients’ overall evaluations of healthcare experiences. Each exogenous construct represents a distinct dimension of healthcare quality: administrative efficiency, interpersonal respect, spatial accessibility, participatory governance, and community engagement. The model is designed to capture the multidimensional nature of trust and satisfaction, with paths hypothesized based on the theory of cooperative healthcare management. Figure 1 depicts the conceptual framework.

### 2.1. Administrative Procedure

Administrative procedure refers to the systematic process by which organizations manage tasks such as providing information, explaining costs, and ensuring smooth operational workflows [8]. Specifically, it corresponds to the structured processes involved in delivering healthcare services, including appointment scheduling, check-in, payment, and coordination of tests and treatments. It reflects the transparency, efficiency, and clarity with which a healthcare organization manages patient-facing operations. Effective administrative procedures can enhance transparency and predictability, which are important factors for fostering trust [15]. Transparent communication and clear information regarding treatment costs and procedures can significantly improve patient confidence in healthcare providers [40,41]. Moreover, when administrative tasks such as check-ins, payments, and treatment processes are streamlined and convenient, patients are more likely to be satisfied with their overall experience [1,42]. Efficient administrative procedures contribute to reducing patient anxiety and increasing their perceived value of the service received. Therefore, this study posits that robust administrative procedures are essential for building trust and satisfaction among patients in healthcare settings.

**H1a.**  *Administrative procedure has a positive impact on trust*.

**H1b.**  *Administrative procedure has a positive impact on satisfaction*.

### 2.2. Courtesy

Courtesy in a healthcare context involves the respectful and considerate treatment of patients by medical staff, encompassing aspects such as empathy, understanding, and attentiveness [11]. When healthcare professionals demonstrate courtesy, it can significantly enhance the perceived quality of care and strengthen the patient’s trust in their healthcare providers [17]. For instance, when doctors show genuine concern for both the physical and mental well-being of their patients, it helps to build a stronger, trust-based relationship [43]. Additionally, courteous interactions contribute to overall patient satisfaction, as patients feel valued and respected during their healthcare experience [18,19]. Positive interpersonal interactions with healthcare providers can lead to higher levels of satisfaction due to the emotional and psychological reassurance they provide [44,45]. Therefore, this study suggests that courtesy plays a critical role in fostering both trust and satisfaction in healthcare settings.

**H2a.**  *Courtesy has a positive impact on trust*.

**H2b.**  *Courtesy has a positive impact on satisfaction*.

### 2.3. Physical Accessibility

Physical accessibility refers to the ease with which patients can access healthcare facilities, including aspects like location convenience, parking availability, and overall facility accessibility [8]. When healthcare services are physically accessible, patients are more likely to have a positive experience, reducing stress and inconvenience associated with reaching the clinic [16]. Easy access to healthcare services ensures timely care and enhances the overall patient experience, contributing to higher satisfaction levels [20,21,22]. Therefore, this study proposes that physical accessibility significantly impacts patient satisfaction.

**H3.**  *Physical accessibility has a positive impact on satisfaction*.

### 2.4. Collaborative Management

Collaborative management in healthcare welfare social cooperative refers to the integration of diverse professional expertise and patient involvement in decision-making processes to improve health outcomes [6]. This approach fosters an environment of transparency and shared responsibility, which can significantly enhance trust among patients, as they feel their perspectives and needs are respected and considered [24]. Moreover, when patients perceive that their care is managed collaboratively, it leads to higher satisfaction, due to the perceived quality and comprehensiveness of the care provided [23]. Therefore, this study suggests that collaborative management significantly impacts both trust and satisfaction in healthcare settings.

**H4a.**  *Collaborative management has a positive impact on trust*.

**H4b.**  *Collaborative management has a positive impact on satisfaction*.

### 2.5. Community Health Relations

Community health relations refer to the collaborative efforts between healthcare providers and various community organizations to improve public health outcomes [6]. Strong community health relations can enhance healthcare delivery by addressing local health needs more effectively and providing comprehensive care [26]. When healthcare providers actively engage with the community and build partnerships, it leads to improved health services, which significantly boosts patient satisfaction [25]. Patients feel more supported and valued when their healthcare providers are involved in community health initiatives, leading to a higher level of satisfaction with their care [46]. Therefore, this study suggests that community health relations significantly impact patient satisfaction.

**H5a.**  *Community health relations have a positive impact on trust*.

**H5b.**  *Community health relations have a positive impact on satisfaction*.

### 2.6. Trust

Trust in healthcare refers to the confidence patients have in their healthcare providers’ competence, reliability, and integrity [47]. When patients trust their healthcare providers, they are more likely to feel secure and valued, leading to a more positive overall experience [48]. Trust can enhance communication, adherence to medical advice, and perceived quality of care, all of which contribute to higher patient satisfaction [49,50]. A strong trust relationship reduces anxiety and increases the likelihood of patients feeling satisfied with their care [3,51]. Therefore, this study posits that trust significantly impacts patient satisfaction.

**H6.**  *Trust has a positive impact on satisfaction*.

## 3. Research Methodology

### 3.1. Measurements

The development of the survey instrument for this study involved several rigorous steps to ensure its validity and reliability, drawing on constructs derived from previously validated studies. To strengthen contextual relevance and content validity, we conducted in-depth interviews with 10 experts affiliated with various health welfare social cooperatives, including former executives, directors, nurses, and individuals with academic backgrounds in medicine, cooperative management, and social welfare. The expert panel was selected based on their extensive experience in cooperative healthcare operations and governance. Through semi-structured interviews, we explored key topics, such as the core values of health welfare social cooperatives, distinctions between cooperative and conventional healthcare services, and the characteristics of quality care in cooperative settings. These discussions informed the refinement and contextualization of subdimensions and items, ensuring their alignment with the cooperative healthcare model. Based on the interview insights, existing measurement items were adapted and new items were developed to reflect the unique service philosophy and operational characteristics of health welfare social cooperatives. This iterative process helped establish the content validity of the instrument prior to its empirical testing.

Table A1 presents a comprehensive list of constructs and associated items used to evaluate various aspects of service quality in health welfare social cooperatives (clinics). The constructs include administrative procedure, courtesy, physical accessibility, collaborative management, community health relations, trust, and satisfaction. Each construct comprises several items designed to assess specific components of the cooperative’s operations and interactions with patients. For instance, the administrative procedure construct, sourced from Frichi, et al. [8], includes items such as the effectiveness of the clinic in providing appointment-related information and explaining treatment costs. The courtesy construct, referencing Debata, et al. [11], evaluates the trustworthiness and care provided by the doctors, including their attention to both mental and physical health. Constructs like physical accessibility [8] and collaborative management [6] assess the logistical convenience of accessing the clinic and the inclusiveness of its management practices, respectively. The community health relations construct examines the cooperative’s engagement with local health initiatives and community organizations [6]. Lastly, the constructs of trust [47] and satisfaction [8,52] measure patients’ confidence in the cooperative’s staff and their overall contentment with the services received, indicating their likelihood of recommending and continuing to use the clinic. The measurement of patient trust and satisfaction in this study was based on a concise, 6-item survey tool—three items for trust and three items for satisfaction. These items were adapted from validated instruments used in prior healthcare research [47,52]. The constructs were designed to capture the overall perception of the patient toward healthcare providers and services in a cooperative setting. While brief, this approach was intentional to reduce respondent burden and improve response quality. Previous studies have shown that short scales, when well-targeted and validated, can effectively measure broad attitudinal constructs without compromising reliability or validity [53].

The items on the survey utilized a five-point Likert scale, ranging from 1 (strongly disagree) to 5 (strongly agree), to measure respondents’ attitudes and perceptions accurately. To further ensure content validity, a pre-test of the instrument was conducted through interviews with experts from the medical cooperative sector. These interviews helped refine the questions to better fit the study’s context and the respondents’ understanding. Following the expert reviews, a pilot test was conducted with a sample of 20 voluntary participants, including members of health welfare social cooperatives and researchers familiar with healthcare and cooperative studies. Participants were asked to complete the survey and provide feedback on item clarity, logical flow, and overall comprehension. Based on their input, several revisions were made to enhance the logical consistency of question order and to eliminate ambiguous or confusing wording. This step helped ensure that the final version of the questionnaire was both understandable and contextually appropriate for the target population.

### 3.2. Data Collection

This study employed a survey methodology, widely justified in research for its effectiveness in collecting large amounts of data in a structured manner. The target sample comprised members of 13 national health welfare social cooperatives, excluding dentists, due to their direct relevance to the study. These 13 cooperatives were selected based on regional diversity and their willingness to participate in the research. The selection aimed to capture a wide geographic distribution across urban and rural areas in South Korea, ensuring variability in cooperative size, structure, and local community engagement. Although the sampling was not random, it was purposive to reflect diverse cooperative environments and enhance the generalizability of findings within the cooperative healthcare context. Eligible participants had to be members for at least six months and have prior experience with medical or traditional Korean medical services. Data collection was conducted both online and offline from 31 March to 14 April 2022. The recruitment process was facilitated through official channels: each cooperative distributed the survey file and online link via email and phone communication to affiliated institutions. The study purpose—supporting the advancement of health welfare social cooperatives—was clearly emphasized to encourage engagement. Participation was entirely voluntary and anonymous, with no financial or material incentives provided. However, many participants actively engaged with the survey, recognizing its potential to benefit the long-term development of their cooperative organizations. Informed consent was obtained from all participants, and data confidentiality was strictly maintained throughout the study.

Out of the total responses collected, the data filtering criteria involved removing entries with missing values or insincere responses, resulting in 658 valid responses being used for analysis. This pre-processing step was necessary to ensure the quality and reliability of the data analyzed in the study. Table 1 provides a breakdown of survey respondents’ demographic and socioeconomic characteristics. It shows gender distribution, with 28.0% male and 71.7% female participants. Age-wise, the majority are in their 50s (51.5%), followed by the 60s (20.7%), and 40s (19.8%). The income range varies with 17.3% earning below 2 million won and smaller segments spanning up to over 700 million won, where 15.0% earn above. Membership duration in the organization ranges widely, with 24.9% being members for 10 years or more. Lastly, 58.8% of participants are actively contributing to regular dues, showing significant engagement.

## 4. Results

This study employed Partial Least Squares Structural Equation Modeling (PLS-SEM) to analyze the data, as this method is particularly effective for complex model testing and theory building in exploratory research [54]. PLS-SEM is preferred in scenarios involving small to medium sample sizes and when the distribution assumptions are not strictly normal [54]. All analyses were conducted using SmartPLS version 4.1.0.2, which provides robust tools for model estimation and evaluation in variance-based SEM. The results confirmed that the theoretical framework was adequately supported by the empirical data.

### 4.1. Common Method Bias (CMB)

To address CMB in this study, a single factor analysis was conducted, revealing that a single factor accounted for 47.963% of the variance, which suggests a potential influence of common method bias. However, the Variance Inflation Factor (VIF) values were examined to further assess multicollinearity among the constructs. All VIF values were well below the recommended threshold of 3.3 [55], indicating an acceptable level of multicollinearity. Specifically, the highest VIF recorded was 2.719 for the relationship between trust and satisfaction, which suggests that the data do not suffer significantly from multicollinearity issues, supporting the validity of the model’s results.

### 4.2. Measurement Model

The measurement model of this study was rigorously evaluated to ensure the reliability and validity of the constructs utilized. Table 2 presents the factor loadings, Cronbach’s Alpha, Composite Reliability (CR), and Average Variance Extracted (AVE) for each construct. The results indicate satisfactory internal consistency for all constructs, as evidenced by Cronbach’s Alpha values which are well above the acceptable threshold of 0.7 for most constructs [56]. Additionally, the AVE values are above 0.5, confirming adequate convergent validity [57], and the CR values exceed the recommended threshold of 0.7, further validating the constructs’ reliability [54].

The Fornell–Larcker criterion, as detailed in Table 3, further justifies the discriminant validity of the constructs, with the square root of AVE for each construct being greater than its correlations with other constructs [57]. This suggests that, despite the HTMT values, each construct shares more variance with its indicators than with other constructs, reinforcing their retention in the study.

### 4.3. Structural Model

The structural model was assessed using 5000 resampling bootstraps, revealing that the model explains 63.1% of the variance in trust and 76.4% in satisfaction. This indicates a strong explanatory power for the relationships between the constructs within health welfare social cooperatives [54]. Table 4 details the results of the SEM test.

## 5. Discussion

The findings indicate that administrative procedure has a significant positive impact on trust but does not significantly affect satisfaction. The positive impact on trust aligns with previous studies emphasizing the importance of efficiency and effectiveness in administrative processes for building patient trust [8,15]. Clear and efficient administrative procedures enhance patients’ confidence in the healthcare system, reinforcing institutional trust. However, the lack of significant impact on satisfaction suggests that, while administrative efficiency builds trust, it may not directly translate into overall satisfaction. This could be due to patients placing higher importance on other factors, such as the quality of care and interpersonal interactions, for their overall satisfaction.

Courtesy shows a strong positive impact on both trust and satisfaction. The significant effect of courtesy on trust reinforces the idea that respectful and empathetic treatment by healthcare staff is vital for building trust [11,17]. Respectful and empathetic communication from healthcare providers strengthens interpersonal trust between patients and providers. When healthcare providers show genuine concern for patients’ well-being, it fosters a trust-based relationship. Furthermore, the positive impact on satisfaction highlights that courteous interactions make patients feel valued and respected, enhancing their overall healthcare experience. Courteous treatment leads to a more positive patient experience, increasing overall satisfaction with healthcare services. This supports the findings of previous research, which noted that positive interpersonal interactions significantly contribute to patient satisfaction [18,19].

The analysis also indicates that physical accessibility positively impacts satisfaction. This finding is consistent with prior research showing that easy access to healthcare services reduces patient stress and enhances the overall experience [16,20]. Convenient access to facilities directly contributes to patient comfort and ease, positively influencing satisfaction levels. This underscores the importance of physical accessibility in healthcare planning and facility management, emphasizing that the ease of reaching a healthcare facility is a critical factor in patient satisfaction [21].

The results reveal that collaborative management does not significantly impact trust. This indicates that other factors, such as direct communication and transparency, play a more important role in building trust than collaborative management alone. Given that the respondents of this study are members of a health welfare social cooperative who have paid contributions and participate in the cooperative’s management, it is possible that their trust is already established through other means of engagement and transparency within the cooperative structure. However, collaborative management positively impacts satisfaction, supporting the notion that involving patients and integrating diverse professional expertise enhances perceived quality and comprehensiveness of care [23,24]. Involving patients in decision-making and cooperative governance fosters a sense of ownership and personal relevance, improving satisfaction. For members of health welfare social cooperatives, their active involvement in the management is likely to have made them more sensitive to the quality of collaborative efforts. This involvement ensures that care delivery is more tailored to their needs and expectations, thereby enhancing overall satisfaction. These findings suggest that, while collaborative management may not directly build trust among cooperative members, it significantly contributes to their satisfaction by improving the quality of care and the patient experience.

Moreover, the results indicate that community health relations have a significant positive impact on trust and satisfaction. Active community engagement by healthcare providers enhances public perception of credibility and commitment, thereby building trust. Further, services aligned with local health needs increase patients’ sense of value and connection, boosting satisfaction. This finding aligns with previous research showing that effective collaboration between healthcare providers and community organizations can enhance service delivery and address local health needs more effectively [6,25]. For the members of health welfare social cooperatives, community health relations are particularly important. When healthcare providers actively engage in community health initiatives, it fosters a supportive environment where patients feel more valued and cared for. This engagement can take various forms, such as partnerships with local organizations, community health education programs, and outreach initiatives, all of which contribute to higher satisfaction levels among cooperative members. For health welfare social cooperatives, community engagement is not just beneficial but integral to their operational model. These cooperatives rely on the active participation and support of their members and the broader community to function effectively.

Trust has the most substantial positive impact on satisfaction among all the factors examined. Trust in both healthcare providers and institutions is a foundational element that drives patient satisfaction in cooperative healthcare models. This significant relationship supports the idea that trust is a fundamental component of patient satisfaction [47,48]. When patients trust their healthcare providers, they are more likely to feel secure, valued, and satisfied with the care they receive. Trust enhances communication, adherence to medical advice, and perceived quality of care, all of which contribute to a more positive overall experience [49,50]. This strong correlation suggests that building and maintaining trust should be a primary focus for healthcare providers aiming to improve patient satisfaction [3].

This study deepens the understanding of trust and satisfaction by empirically identifying which factors most significantly influence these outcomes within the unique context of health welfare social cooperatives. By integrating organizational (administrative procedure, collaborative management), relational (courtesy, trust), and systemic (community health relations, physical accessibility) predictors into a comprehensive model, the findings highlight how trust functions both as an outcome and as a powerful driver of satisfaction. This contributes to cooperative healthcare literature by demonstrating that beyond clinical care, patient-centered administrative efficiency, participatory management, and community responsiveness are essential for cultivating trust and sustaining satisfaction.

## 6. Conclusions

### 6.1. Implications for Researchers

This study offers significant theoretical contributions to the understanding of factors influencing patient trust and satisfaction within health welfare social cooperatives. By examining the roles of administrative procedures, courtesy, physical accessibility, collaborative management, and community health relations, this research expands on existing literature and provides new insights into the dynamics of patient–provider interactions.

Firstly, the study identifies the distinct role of administrative procedures in building trust, but not satisfaction. While previous research has highlighted the importance of efficient administrative processes in enhancing trust [8,15], this study clarifies that such procedures do not necessarily translate to overall satisfaction. This finding suggests that, although clear and efficient administrative practices are important for establishing trust, patients may prioritize other aspects, such as interpersonal interactions and quality of care, for their satisfaction [1]. Scholars should consider investigating other factors that could bridge this gap, potentially exploring how administrative transparency interacts with personal care quality to enhance both trust and satisfaction.

Secondly, this study stresses the strong impact of courtesy on both trust and satisfaction. Previous studies have emphasized the importance of respectful and empathetic treatment in building trust [11,17], but this research extends those findings by demonstrating that courtesy also significantly enhances satisfaction. This dual impact highlights the critical role of interpersonal interactions in healthcare settings. While earlier studies acknowledged the importance of courtesy, they did not fully explore its comprehensive effects on patient satisfaction. Future research should delve deeper into the specific elements of courteous behavior that most effectively enhance both trust and satisfaction, potentially examining cultural and contextual differences in patient expectations and perceptions.

Furthermore, the study reveals the differential impacts of collaborative management on trust and satisfaction. Contrary to prior studies suggesting that collaborative management universally enhances trust [6], this research shows that, while it significantly boosts satisfaction, it does not directly influence trust among members of health welfare social cooperatives. This discrepancy indicates that trust may be established through other mechanisms within cooperative settings, such as transparency and direct communication, rather than collaborative management alone. This insight challenges existing theories and suggests that future studies should investigate the unique contexts and factors that build trust in cooperative healthcare models. Scholars could explore how different forms of patient engagement and transparency practices interact with collaborative management to enhance both trust and satisfaction.

### 6.2. Implications for Practitioners

This research provides actionable insights for healthcare providers and cooperative managers. Firstly, the study highlights the importance of efficient administrative procedures in building trust. Healthcare providers and cooperative managers should focus on streamlining administrative processes to enhance transparency and predictability. Implementing clear communication channels for appointment scheduling, treatment cost explanations, and procedural updates can significantly build patient trust [58]. Health welfare social cooperatives can adopt digital platforms to automate these processes, reducing wait times and ensuring that patients receive timely information. Additionally, training administrative staff to handle inquiries efficiently and empathetically can further improve patient experiences and foster trust.

Secondly, the research maintains the dual impact of courtesy on both trust and satisfaction. Practitioners should prioritize cultivating a culture of respect and empathy among healthcare staff. Training programs focusing on soft skills, such as active listening, empathetic communication, and patient-centered care, can be highly effective [59,60]. Healthcare providers should be encouraged to take the time to listen to patients’ concerns and respond with genuine care and understanding [61]. This approach not only builds trust but also significantly enhances patient satisfaction by making them feel valued and respected. Health welfare social cooperatives can implement regular workshops and role-playing scenarios to continuously improve staff–patient interactions.

Furthermore, the study’s findings on physical accessibility suggest that improving logistical aspects of healthcare facilities can enhance patient satisfaction. Healthcare providers should ensure that their facilities are easily accessible, with adequate parking and convenient locations [62]. Cooperatives can conduct accessibility audits to identify and address potential barriers that patients might face when accessing care. Simple measures, such as clear signage, ramps for wheelchair access, and comfortable waiting areas, can make a significant difference in patient satisfaction. Additionally, offering services such as home visits or telehealth options can cater to patients with mobility issues, further improving their access to care.

Moreover, the study reveals that collaborative management positively impacts satisfaction, suggesting that involving patients in decision-making processes can enhance their overall experience. Practitioners should actively engage cooperative members in governance and care planning activities. For example, establishing patient advisory councils or committees where members can voice their opinions and contribute to decision-making can make them feel more invested in their care. This participatory approach ensures that healthcare services are tailored to the specific needs and preferences of the cooperative members, thereby increasing satisfaction.

Finally, the significant positive impact of community health relations on satisfaction underlines the importance of community engagement in healthcare. Healthcare providers should build strong partnerships with local organizations and participate in community health initiatives. For instance, collaborating with community centers, schools, and local businesses to provide health education programs, screenings, and preventive care can foster a supportive environment. Health welfare social cooperatives can organize regular community events and health fairs to promote wellness and strengthen ties with the community. This proactive approach not only enhances patient satisfaction but also contributes to the overall well-being of the community.

### 6.3. Limitation and Further Research

This study has several limitations. Firstly, the sample is limited to members of a health welfare social cooperative, which may not be representative of the broader population. Future research should include diverse healthcare settings to enhance generalizability. Secondly, the study relies on self-reported data, which can be subject to biases. Incorporating objective measures or third-party evaluations could provide more robust findings. Lastly, this study does not explore the potential moderating effects of demographic variables such as age or socioeconomic status. Future research should examine these factors to understand their influence on the relationships between administrative procedures, courtesy, physical accessibility, collaborative management, community health relations, trust, and satisfaction.

## Figures and Tables

**Table 1:** Demographic Features of Respondents.

Category	Subject	Frequency	Percentage
Gender	Male	184	28.0%
	Female	472	71.7%
	No Response	2	0.3%
Age	20s and younger	5	0.8%
	30s	35	5.3%
	40s	130	19.8%
	50s	339	51.5%
	60s	136	20.7%
	70s and older	13	2.0%
Income (million KRW)	<2	114	17.3%
	2 to less than 3	112	17.0%
	3 to less than 4	106	16.1%
	4 to less than 5	76	11.6%
	5 to less than 6	84	12.8%
	6 to less than 7	67	10.2%
	7 or more	99	15.0%
Membership Duration	Less than 3 years	132	20.1%
	3 to less than 6 years	193	29.3%
	6 to less than 10 years	169	25.7%
	10 years or more	164	24.9%
Regular Contribution Participation	Not Participation	271	41.2%
	Participating	387	58.8%

**Table 2:** Factor Analysis and Reliability.

Construct	Item	Mean	St. Dev.	Factor Loading	Cronbach’s Alpha	CR (rho_a)	CR (rho_c)	AVE
Administrative Procedure	ADM1	4.229	0.726	0.920	0.847	0.856	0.908	0.766
	ADM2	4.122	0.803	0.867				
	ADM3	4.213	0.706	0.838				
Courtesy	COU1	4.485	0.624	0.851	0.831	0.835	0.899	0.747
	COU2	4.229	0.752	0.843				
	COU3	4.439	0.607	0.899				
Physical Accessibility	PHY1	4.009	0.916	0.771	0.655	0.779	0.805	0.585
	PHY2	2.971	1.086	0.607				
	PHY3	4.144	0.871	0.889				
Co-operation	CMA1	4.099	0.713	0.779	0.777	0.779	0.871	0.693
	CMA2	3.976	0.838	0.849				
	CMA3	4.090	0.764	0.868				
Community Health Relations	CHR1	4.173	0.723	0.865	0.860	0.862	0.915	0.781
	CHR2	4.401	0.666	0.894				
	CHR3	4.295	0.692	0.892				
Trust	TRU1	4.441	0.622	0.907	0.906	0.909	0.941	0.843
	TRU2	4.327	0.649	0.905				
	TRU3	4.380	0.618	0.941				
Satisfaction	SAT1	4.444	0.639	0.913	0.876	0.882	0.923	0.800
	SAT2	4.477	0.626	0.883				
	SAT3	4.301	0.662	0.888				

**Table 3:** Fornell–Larcker Scale Results.

Construct	1	2	3	4	5	6	7
1. Administrative Procedure	0.875						
2. Courtesy	0.613	0.864					
3. Physical Accessibility	0.506	0.399	0.765				
4. Collaborative Management	0.515	0.557	0.410	0.833			
5. Community Health Relations	0.547	0.614	0.394	0.592	0.884		
6. Trust	0.637	0.733	0.364	0.533	0.646	0.918	
7. Satisfaction	0.582	0.705	0.419	0.557	0.639	0.857	0.894

Note: Diagonal elements are the square root of AVE.

**Table 4:** Test Results.

H	Predictor	Outcome	*β*	*SE*	*t*	*p*	Result
H1a	Administrative Procedure	Trust	0.227	0.040	5.612	<0.01	Supported
H1b	Administrative Procedure	Satisfaction	−0.047	0.034	1.382	0.083	Not Supported
H2a	Courtesy	Trust	0.427	0.039	10.945	<0.01	Supported
H2b	Courtesy	Satisfaction	0.106	0.039	2.718	0.003	Supported
H3	Physical Accessibility	Satisfaction	0.090	0.027	3.293	<0.01	Supported
H4a	Collaborative Management	Trust	0.037	0.033	1.120	0.131	Not Supported
H4b	Collaborative Management	Satisfaction	0.073	0.027	2.755	0.003	Supported
H5a	Community Health Relations	Trust	0.238	0.043	5.513	<0.01	Supported
H5b	Community Health Relations	Satisfaction	0.076	0.031	2.429	0.008	Supported
H6	Trust	Satisfaction	0.688	0.038	18.342	<0.01	Supported

## Data Availability

The data used in this study are available from the corresponding authors.

## Appendix A

**Table A1:** List of Constructs and Items.

Construct	Item	Description	Source
Administrative Procedure	ADM1	This health welfare social cooperative (clinic) is good at providing information about waiting or starting an appointment.	Frichi, et al. [8]
	ADM2	This health welfare social cooperative (clinic) explains the cost of treatment in detail.	
	ADM3	This health welfare social cooperative (clinic) has a convenient process for checking in, paying, and receiving treatment and tests.	
Courtesy	COU1	I trust the diagnosis and treatment decisions of the doctors at this health welfare social cooperative (clinic).	Debata, et al. [11]
	COU2	The doctor at this health welfare social cooperative (clinic) cares about my mental health as well as my physical health.	
	COU3	My doctor understands my explanations and questions.	
Physical Accessibility	PHY1	This health welfare social cooperative (clinic) is accessible.	Frichi, et al. [8]
	PHY2	This health welfare social cooperative has convenient parking.	
	PHY3	This health welfare social cooperative is easy to get to.	
Collaborative Management	CMA1	I think this health welfare social cooperative involves people from different fields in its operations.	Kim and Park [6]
	CMA2	I feel that I am the owner of this health welfare social cooperative (clinic).	
	CMA3	I think this health welfare social cooperative is co-owned by all members.	
Community Health Relations	CHR1	I think this health welfare social cooperative (clinic) has a good working relationship with various organizations in the community to improve the health of its residents.	Kim and Park [6]
	CHR2	I think this health welfare social cooperative (clinic) does a good job of providing medical support for the needs of the community (home visits, medical support for the socially disadvantaged, etc.)	
	CHR3	I think this health welfare social cooperative (clinic) makes an effort to identify the health needs of the local population and community.	
Trust	TRU1	I have overall trust in the doctors in this health welfare social cooperative (clinic).	Chang, et al. [47]
	TRU2	I have overall confidence in the staff at this health welfare social cooperative.	
	TRU3	I have overall confidence in this health welfare social cooperative (clinic).	
Satisfaction	SAT1	I would recommend using this health welfare social cooperative to someone I know.	Frichi, et al. [8] Zhou, et al. [52]
	SAT2	I will use this health welfare social cooperative (clinic) in the future.	
	SAT3	I am satisfied overall with this health welfare social cooperative (doctor’s office).	
